# Vitamin D Status during Pregnancy versus the Anthropometric Parameters of Two- and Four-Year-Olds: A Pilot Study

**DOI:** 10.3390/nu14020254

**Published:** 2022-01-07

**Authors:** Regina Ewa Wierzejska, Barbara Katarzyna Wojda

**Affiliations:** Department of Nutrition and Nutritional Value of Food, National Institute of Public Health NIH—National Research Institute, Chocimska St. 24, 00-791 Warsaw, Poland; bwojda@pzh.gov.pl

**Keywords:** vitamin D, pregnancy, neonates, anthropometric parameters, children

## Abstract

Apart from being associated with a well-documented risk for adverse pregnancy outcomes, maternal deficiency of vitamin D may also negatively affect the physical development of their children. The aim of the study was to evaluate the relationship between maternal as well as umbilical cord blood levels of vitamin D and the weight and height values of two- and four-year-olds. The study was conducted in a group of 52 ‘mother–child’ pairs. On the day of the delivery, total 25(OH)D concentration in blood was measured using immunological tests (LIAISON). Weight and height values were obtained from the database of routine health checks for children aged two and four, which are obligatory in Poland. Multiple regression analysis was used for statistical analysis. No association was detected between maternal-neonatal concentrations of vitamin D and weight and height values of the investigated two- and four-year-olds despite extreme differences in maternal (4.0–37.7 ng/mL) and neonatal (5.9–46.6 ng/mL) concentrations and the fact that vitamin D deficiency was detected in almost 54% of the mothers and 37% of the newborns. Therefore, no relationship between maternal-fetal vitamin D concentrations and the anthropometric parameters of the investigated children up to the age of four was found.

## 1. Introduction 

Vitamin D deficiency is a global public health problem which also affects pregnant women [1,2,3]. Although there is no consensus in academia on the nomenclature and the criteria concerning vitamin D levels, most experts believe that a concentration 25(OH)D of <20 ng/mL (<50 nmol/L) should be defined as ‘deficiency’ [4,5]. With such threshold values, vitamin D deficiency has been detected in approximately 57% of pregnant women in Europe, including 31–50% in Poland, as well as 64% in North America, and 87% in South-East Asia. Supply of vitamin D to the fetus is closely correlated to maternal levels, and cord blood vitamin D deficiency has been found in 73% of the newborns in Europe, 30% in North America, and 96% in South-East Asia [4,6,7,8,9]. Prevalent vitamin D deficiencies are due to the fact that it is not easy to meet the requirements because of scarcity of the component in food. The only natural, high source of vitamin D are fatty fish, but their consumption is generally low and does not provide sufficient amounts of this vitamin. Eggs are another significant dietary source of vitamin D, while milk and milk products are very low in this component [10,11]. In view of the small number of food products containing vitamin D, it is estimated that the diet covers at most 20% of the requirement [12]. In light of the meta-analysis of studies on the intake of this vitamin in developed countries, the median intake by pregnant women is 3.3 µg daily [13] and similar results were obtained in Polish studies which were not included in the above-mentioned analysis (2–3 µg) [14]. With a requirement of 15 µg/day, such an intake is obviously by far deficient [15,16]. The situation is no better in the case of children. For example, the intake of vitamin D by children aged 1.5–3.5 years in the UK is on average about 1.7 µg/day [10], and in Poland, according to several studies, children aged 1–6 years take on average from 0.6 µg to 3.5 µg [17,18]. Higher intakes of vitamin D are found in countries such as the USA, Canada and Finland, where a programme of food enrichment with this vitamin (mainly milk) was launched, and there the average intake by children is 4.4–5.9 µg [10]. In summer, there is a chance to improve the body’s supply of vitamin D as a result of its dermal synthesis, but changes in lifestyle, including long periods spent indoors, mean that blood concentrations of 25(OH)D often do not reach the recommended values [19]. In the case of young children there is also the issue of protecting them from excessive sunlight, and in recent years this has been particularly important because of recurrent ‘heat waves’. Therefore, because of vitamin D deficiency in the diet and in the body, many countries recommend widespread supplementation of this component [20].

Low maternal vitamin D concentration increases the risk for unfavorable course of pregnancy, including pre-eclampsia, diabetes, preterm labor, hypocalcemia in the newborn, and birth of a small-for-gestational-age infant [21,22,23]. According to the latest studies, the lowest required vitamin D concentration, which would suffice to eliminate high risk for those complications, has been estimated at 15 ng/mL [24]. As far as the relationship between maternal vitamin D concentration and anthropometric parameters in the newborn is concerned, the literature offers ambiguous findings [25,26], but 15 ng/mL are also believed to be the threshold value. According to a meta-analysis of the observational studies, the concentration of <15 ng/mL results in a birthweight decrease by 131 g, with no effect on the neonatal length and head circumference [27].

Maternal vitamin D deficiency may also affect the development of the child later in life [23,28]. Impaired immune system, resulting in higher risk for respiratory infections and asthma, lower bone mineral density, higher risk for type 1 diabetes, and sepsis in the newborn are among the most common consequences of vitamin D deficiency in utero reported in the literature [29,30,31,32,33]. The exact mechanism of the relationship between low vitamin D concentration and elevated risk for an abnormal pregnancy course and infant development remains to be fully elucidated, but it is believed to be rooted in poor vascularization of the placenta, abnormal immunological response between the placenta and the fetus, including activation of proinflammatory cytokines, compromised placental defense against the entry of bacteria, and impaired glucose tolerance [34,35,36].

The number of studies about the link between physical development of the child and maternal vitamin D deficiency is limited, and those available offer conflicting results [28,37,38]. In our previous study on vitamin D, the concentration of <20 ng/mL was detected in as many as 50% of the mothers during the perinatal period and 28% of their newborns [39]. Therefore, it seemed prudent to conduct further studies to determine whether vitamin D concentration in pregnancy might affect the anthropometric parameters of the children during the first years of their life.

The aim of the study was to evaluate a possible relationship between maternal and cord blood vitamin D concentrations and the weight/height of children at the ages of 2 and 4.

## 2. Material and Methods

### 2.1. Study Design

**Figure 1:**
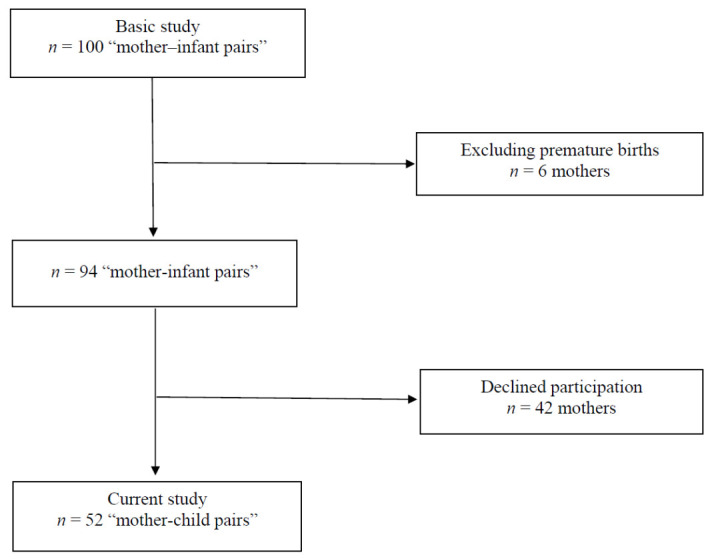
The process of recruiting study participants.

The study was conducted among children born to mothers who had given birth at the Department of Obstetrics, Women’s Diseases and Gynecological Oncology of the Medical University of Warsaw, between 2014 and 2015, and had participated in the earlier study (mother-neonate pairs) on the maternal and cord blood vitamin D concentrations. Out of 100 participants, the current study was conducted in a subgroup of 52 mother-child pairs. All children were born at term. The process of recruiting study participants is presented in Figure 1.

The Ethics Committee of the Institute of Food and Nutrition approved of the study (No. 10/162/KB/2014). The characteristics of the women, newborns, and children are presented in Table 1.

### 2.2. Laboratory Analysis and Data Collection

Maternal blood was collected after admission to the delivery ward (when blood was taken for diagnostic purposes), and cord blood was collected at delivery. Total 25(OH)D [25(OH)D2 and 25(OH)D3] concentrations were measured in the blood using immunological tests (LIAISON^®^ 25 OH Vitamin D TOTAL Assay; DiaSorin Inc, Stillwater, OK, USA). A detailed description of the method used to measure vitamin D concentrations was described in our previous article on the possible relationship between vitamin D levels and the anthropometric parameters of the newborns [39]. The concentration of <20 ng/mL was defined as maternal/cord blood vitamin D deficiency [4,5,40]. Based on direct interview with patients, conducted face-to-face during their hospital stay, among other things, vitamin D intake during pregnancy was estimated using the validated Food Frequency Questionnaire and using the “Photo Album of Meals and Products”. In addition, data on calcium intake, caffeine intake, and lifestyle of pregnant women were collected.

Data about weight/height of the children at the ages of 2 and 4 were collected using a telephone interview between the mothers and a pediatrician from the hospital where the women give birth. In accordance with the standards for child health care in Poland, children at the ages of 2 and 4 are required to undergo routine health checks and the data are recorded in their personal health records (PHR). During the telephone interview, the mothers supplied information about the weight/height values using the PHR. The time range for the routine health checks of the 2- and 4-year-olds was from 1 week before to 3 weeks after the child turned 2 and from 2 weeks before to 6 weeks after the child turned 4, respectively. The weight and the height were measured with the accuracy of 100 g and 1.0 cm, respectively.

### 2.3. Statistical Analysis

Normal distribution of all studied parameters was checked using the Kolmogorov–Smirnov test. Simple linear regression was used to analyze venous versus cord blood vitamin D concentration. An adjusted multiple regression analysis was used to investigate a possible relationship between selected baseline characteristics (serum vitamin D concentration in maternal and umbilical cord blood, gestational age, neonatal weight, pre-pregnancy BMI, weight gain during pregnancy, gravidity, maternal age, vitamin D and calcium consumption, smoking, caffeine consumption, sex of the neonate) and weight and height of the children at the ages of 2 and 4. The *p*-value of < 0.05 was considered statistically significant.

## 3. Results

In the group of mothers and newborns studied, vitamin D deficiency (25(OH)D concentration <20 ng/mL) was present in 53.9% of mothers and 36.5% of newborns. There was a strong correlation between vitamin D concentration in maternal venous blood and newborns’ cord blood, and in all of the investigated cases 25(OH)D concentration in cord blood was higher than in maternal blood (mean 25.4 ± 10.8 vs. 18.3 ± 8.0 ng/mL). With a 1 ng/mL increase in the maternal levels, umbilical cord blood vitamin D concentration increases by approximately 1.19 ng/mL (95% CI = [1.07, 1.30] (R2 = 0.71, *p* < 0.0001).

No relationship was found between maternal-neonatal concentrations of vitamin D and weight/height values of the investigated 2- and 4-year-olds, even despite extreme differences in maternal and neonatal concentrations (4.0–37.7 ng/mL and 5.9–46.6 ng/mL in the mothers and the newborns, respectively) (Table 2, Table 3, Table 4 and Table 5). In addition, no association was found between weight/height values of the 2-year-olds and any of the investigated parameters (Table 2 and Table 3). As for the 4-year-olds, a relationship was found between their weight at the age of 2 and 4. Children who at the age of 2 were heavier than their peers (by 1 kg) were also heavier (by 0.76 kg) at the age of 4 (Table 4).

No statistically significant differences were found between the number of children <3 centile or >90 centile, either for weight or height, with vitamin D levels of <20 ng/mL vs. ≥20 ng/mL.

## 4. Discussion

The correlation found between vitamin D concentrations in maternal and neonatal cord blood is well documented in the literature. Most of the studies, such as ours, indicate that the concentration of this vitamin in cord blood is higher than in mothers’ blood [41,42,43]. As was the case in our previous study on the anthropometric parameters of the newborns and vitamin D [39], in the present study, we also found no relationship between maternal-neonatal concentrations of vitamin D and weight/height values of the children up to the age of 4, despite a wide range of detected concentrations for women and neonates and maternal deficiency in over half of the women. Our findings are consistent with the majority of the reports in the literature worldwide. In a study from China, no relationship between vitamin D levels and weight, height, and BMI of children up to the age of 3 was found, regardless of the fact that vitamin D concentration of <20 ng/mL was found in 90% of the mothers [37]. In a study from Spain, no link between such vitamin D concentration and the risk for overweight in 4-year-olds was detected [44], while in a study from Holland, maternal vitamin D concentration was not related to the height of their children aged from 5 to 6 years [45]. In a multi-centre cohort study from US, very low concentration of vitamin D in pregnant women (<12 ng/mL), which was associated with deterioration of the anthropometric parameters in their newborns, had no subsequent consequences for weight values of the children at the age of 1, whereas lower height values persisted up to 12 months of their life [28]. On the other hand, in a study from Finland, high maternal concentration of vitamin D (>50 ng/mL) was associated with delayed growth in their newborns, diagnosed at 6 and 12 months [46].

As far as cord blood concentration of vitamin D is concerned, the available studies—albeit limited in number—found no relationship with the physical development of children in the first years of their life. Such analyses have been carried in Australia—twice, in children aged 1.5 and 4 years [47], in Denmark—ages: 3 months, 1.5 and 3 years [48], and in China—ages: from 6 months to 2 years [34].

The literature offers several attempts to determine whether vitamin D supplementation during pregnancy may be in any way associated with the anthropometric parameters in young children. In a randomized study by Roth et. al., children at the age of 12 months, born to mothers whose weekly supplementation with vitamin D in the third trimester was 35,000 IU, were taller than controls [49], whereas in a study by Brustad et al., supplementation during pregnancy with 2800 IU/day, as compared to 400 IU, did not affect the anthropometric parameters in children aged 6 years [31]. In light of the meta-analysis of randomized studies, maternal vitamin D supplementation is associated with higher weight/height of their children during the first year of life [50]. Importantly, the attitudes towards vitamin D supplementation during pregnancy vary across countries. According to the latest (2016) global consensus of various experts and associations, supplementation with 600 IU/day is recommended in pregnancy [38], but the 2020 World Health Organization (WHO) update specified that supplementation should be recommended only to women with vitamin D deficiency and that 200 IU/day will suffice [51]. The Polish Society of Gynecologists and Obstetricians guideline linked the dose of vitamin D with BMI: 1500–2000 IU/day are recommended to women with normal weight, whereas as many as 4000 IU/day may be advised to obese women, under supervision of a physician [52]. In our present study, almost all women reported vitamin-mineral supplementation, with low dose of vitamin D (mean 400 IU/day), and only 13% of the participants used single vitamin D preparations (mean 1000 IU/day). As studies prove, low doses of vitamin D do not sufficiently increase its concentration in blood of pregnant women [6,53].

The relationship between the weight of the 2-year-olds and the 4-year-olds from our study should be approached with caution as such lifestyle factors as diet or genetic predisposition were not analyzed. In addition, our study has several limitations, chief among them a small sample size, as only half of the mothers from the previous vitamin D study consented to participate. Furthermore, we have no data on infant nutrition and vitamin D supplementation. From information collected from mothers we know that 98% of children were breastfed, but the period of breastfeeding was very broad (from 1 month to 18 months) and in retrospect mothers were not able to determine exactly how long the children were exclusively breastfed and from when they were fed in a combined way (breastfeeding and formula). These data are very important because breast milk contains negligible amounts of vitamin D, whereas formula milk produced for infants is enriched with vitamin D and drinking 800–1000 mL of such milk per day covers the needs of infants. With regard to supplementation, it is important whether the children born with normal and with low concentrations of vitamin D received the same dose of this vitamin at identical time intervals. Another limitation of the study is the lack of information on the exposure of children to sunlight around midday, when vitamin D is formed in the skin. In addition, it is vital to bear in mind that in our study, the analysis of vitamin D level was conducted on the day of the delivery, which does not signify that the concentration was typical for the entire course of pregnancy or at least its significant part. Lack of unified measurements—the height and the weight were measured in various health clinics, not in one center—constitutes yet another limitation of the present study. Even though the equipment in those clinics and the measurement protocols are regulated by law [54], that fact might have affected the accuracy of the obtained values. The differences in routine health check timelines for 2- and 4-year-olds seem insignificant—no more than 8 weeks- but need to be listed among the possible limitations as well. The authors are aware of all the above-mentioned limitations but feel confident that this pilot study in Poland and the experience may be valuable resources to be used in further research, on a larger sample size.

## 5. Conclusions

In the study group of mothers and children, it was not shown that the supply of vitamin D during foetal life is related to the somatic development of young children. However, future studies should take into account the children’s diet and vitamin D supplementation, including the intake of not only single preparations of this vitamin, but also recently popular vitamin-mineral supplements. Consideration of nutritional factors is very important because the potentially negative effects of deficiency of this vitamin during pregnancy on children’s physical development are perhaps corrected by adequate vitamin D supply during the first years of life.

## Figures and Tables

**Table 1:** Maternal, neonatal and children characteristics.

**Mothers:**	
Number of women, *n* (%)	52 (100)
Age (in years) mean ± SD	30.2 ± 5.0
Education, *n* (%)	
higher	36 (69.3)
other	16 (30.7)
Gravidity, *n* (%)	
primiparas	24 (46.2)
multiparas	28 (53.8)
Pre-pregnancy maternal BMI, mean ± SD	23.1 ± 4.2
Weight gain during pregnancy, *n* (%)	
low	10 (19.2)
normal	22 (42.3)
excessive	20 (38.5)
Gestational diabetes, *n* (%)	6 (11.5)
Smoking during pregnancy, *n* (%)	8 (15.4)
Professionally active during pregnancy, *n* (%)	31 (59.6)
Supplementation with vitamin/mineral preparations, *n* (%)	51 (98.1)
Supplementation with single-component vitamin D preparations, *n* (%)	7 (13.4)
Daily vitamin D consumption—from diet and supplements (µg)	
median (min-max)	19.3 (1.2–73.7)
Daily calcium consumption—from milk and dairy products (mg)	
mean ± SD	689 ± 455
Daily caffeine consumption—from coffee, tea, and energy drinks (mg) median (min-max)	50 (0–203)
Maternal serum vitamin D concentrations (ng/mL) mean ± SD	18.3 ± 8.0
**Newborns**:	
Gestational age (weeks) mean ± SD	39.0 ± 1.9
Sex of the newborn	
male, *n* (%)	25 (48.1)
female, *n* (%)	27 (51.9)
Neonatal weight (g) mean ± SD	3512 ± 578
Low birth weight (<2500 g), *n* (%)	3 (5.8)
Macrosomia (>4000 g), *n* (%)	11 (21.2)
Neonatal length (cm) mean ± SD	55.6 ± 3.0
Apgar score (points) mean ± SD	9.9 ± 0.1
Neonatal head circumference (cm) mean ± SD	34.7 ± 1.5
Neonatal chest circumference (cm) mean ± SD	33.9 ± 2.3
Cord blood vitamin D concentrations (ng/mL) mean ± SD	25.4 ± 10.8
**Children at the age of 2:**	
weight (kg) mean ± SD	11.3 ± 1.1
number of children with weight of <3 centile, *n* (%)	2 (3.7)
number of children with weight of >90 centile, *n* (%)	0 (0)
height (cm) mean ± SD	89.5 ± 2.6
number of children with height of <3 centile, *n* (%)	0 (0)
number of children with height of >90 centile, *n* (%)	15 (28.8)
**Children at the age of 4:**	
weight (kg) mean ± SD	15.7 ± 1.3
number of children with weight of <3 centile, *n* (%)	2 (3.7)
number of children with weight of >90 centile, *n* (%)	1 (1.9)
height (cm) mean ± SD	103.5 ± 5.2
number of children with height of <3 centile, *n* (%)	3 (5.8)
number of children with height of >90 centile, *n* (%)	7 (13.5)

**Table 2:** Relationship between selected parameters and weight at the age of 2.

	Regression Beta Coefficient (SE)	95% CI	*p*-Value
Maternal serum vitamin D concentration	0.07 (0.543)	−1.04; 1.17	0.8990
Cord blood vitamin D concentration	−0.32 (0.552)	−1.44; 0.81	0.5682
Neonatal weight	0.00 (0.001)	−0.00; 0.00	0.2675
Pre-pregnancy body mass index:			
underweight	−0.06 (0.664)	−1.41; 1.29	0.9317
overweight/obesity	0.26 (0.732)	−1.23; 1.75	0.7280
Low weight gain vs. normal gain	0.07 (0.594)	−1.14; 1.28	0.9061
Excessive weight gain vs. normal gain	−0.01 (0.626)	−1.28; 1.27	0.9893
Gestational age	0.62 (0.158)	−0.26; 0.39	0.6974
Smoking	0.45 (0.594)	−0.76; 1.66	0.4517
Maternal age	0.05 (0.420)	−0.80; 0.91	0.9000
Gravidity	−0.53 (0.394)	−0.86; 0.75	0.8938
Caffeine consumption	−0.01 (0.004)	−0.01; 0.00	0.1630
Vitamin D consumption	0.01 (0.001)	−0.01; 0.03	0.2033
Calcium consumption	0.00 (0.000)	−0.00; 0.00	0.8221
Sex	0.42 (0.419)	−0.43; 1.27	0.2297

**Table 3:** Relationship between selected parameters and height at the age of 2.

	Regression Beta Coefficient (SE)	95% CI	*p*-Value
Maternal serum vitamin D concentration	0.66 (1.279)	−1.95; 3.26	0.6115
Cord blood vitamin D concentration	−0.72 (1.301)	−3.36; 1.94	0.5862
Neonatal weight	0.00 (0.001)	−0.00; 0.00	0.6790
Pre-pregnancy body mass index:			
underweight	−2.22 (1.563)	−5.41; 0.96	0.1655
overweight/obesity	−1.94 (1.723)	−5.46; 1.57	0.2671
Low weight gain vs. normal gain	0.78 (1.398)	−2.05; 3.65	0.5727
Excessive weight gain vs. normal gain	1.42 (1.475)	−1.58; 4.42	0.3433
Gestational age	0.24 (0.373)	−0.52; 0.99	0.5283
Smoking	−1.04 (1.399)	−3.89; 1.81	0.4615
Maternal age	0.03 (0.989)	−1.99; 2.04	0.9791
Gravidity	−1.15 (0.929)	−3.04; 0.74	0.2244
Gestational diabetes	−0.07 (1.522)	−3.17; 3.03	0.9630
Caffeine consumption	−0.02 (0.009)	−0.04; 0.00	0.0901
Vitamin D consumption	−0.01 (0.021)	−0.05; 0.03	0.6663
Calcium consumption	−0.00 (0.001)	−0.00; 0.00	0.6150
Sex	1.17 (0.986)	−0.83; 3.18	0.2428

**Table 4:** Relationship between selected parameters and weight at the age of 4.

	Regression Beta Coefficient (SE)	95% CI	*p*-Value
Weight at the age of 2	0.76 (0.153)	0.44; 1.07	0.0001
Maternal serum vitamin D concentration	0.58 (0.469)	−0.37; 1.54	0.2250
Cord blood vitamin D concentration	0.12 (0.480)	−0.86; 1.10	0.8017
Neonatal weight	0.00 (0.000)	−0.00; 0.00	0.5696
Pre-pregnancy body mass index:			
underweight	0.75 (0.574)	−1.42; 1.92	0.1993
overweight/obesity	0.52 (0.634)	−0.77; 1.82	0.4155
Low weight gain vs. normal gain	−0.91 (0.513)	−1.24; 0.86	0.7110
Excessive weight gain vs. normal gain	−0.61 (0.542)	−1.71; 0.49	0.2703
Gestational age	0.02 (0.137)	−0.26; 0.31	08814
Smoking	0.29 (0.518)	−0.76; 1.35	0.5687
Maternal age	−0.39 (0.363)	−1.13; 0.35	0.2870
Gravidity	−0.03 (0.341)	−0.73; 0.66	09208
Caffeine consumption	−0.02 (0.003)	−0.01; 0.00	0.4480
Vitamin D consumption	0.00 (0.007)	−0.01; 0.02	0.9105
Calcium consumption	−0.00 (0.000)	−0.00; 0.00	0.3271
Sex	−0.30 (0.367)	−1.05; 0.44	0.4107

**Table 5:** Relationship between selected parameters and height at the age of 4.

	Regression Beta Coefficient (SE)	95% CI	*p*-Value
Height at the age of 2	0.47 (0.308)	–0.16; 1.10	0.1361
Maternal serum vitamin D concentration	3.75 (2.237)	–0.81; 8.32	0.1038
Cord blood vitamin D concentration	0.19 (2.279)	–4.45; 4.83	0.9333
Neonatal weight	0.00 (0.002)	–0.00; 0.01	0.0582
Pre-pregnancy body mass index:			
underweight	2.73 (2.809)	–2.99; 8.46	0.3385
overweight/obesity	3.33 (3.063)	–2.91; 9.58	0.2850
Low weight gain vs. normal gain	1.85 (2.450)	–3.15; 6.84	0.4577
Excessive weight gain vs. normal gain	–1.07 (2.607)	–6.39; 4.24	0.6831
Gestational age	–0.71 (0.654)	–2.04; 0.63	0.2873
Smoking	0.55 (2.460)	–4.47; 5.57	0.8244
Maternal age	–1.68 (1.725)	–5.20; 1.84	0.3379
Gravidity	1.06 (1.657)	–11.07; −0.24	0.5275
Gestational diabetes	–5.66 (2.654)	–3.17; 3.03	0.4188
Caffeine consumption	0.00 (0.018)	–0.03; 0.04	0.7561
Vitamin D consumption	–0.00 (0.036)	–0.08; 0.07	0.9343
Calcium consumption	–0.00 (0.001)	–0.00; 0.00	0.6920
Sex	–2.96 (1.756)	–6.54; 0.61	0.1029

## Data Availability

Not applicable.
